# Anticoagulant-free venovenous extracorporeal membrane oxygenation for diffuse alveolar hemorrhage with bowel bleeding caused by antineutrophil cytoplasmic antibody-associated vasculitis: A case report

**DOI:** 10.1016/j.rmcr.2021.101513

**Published:** 2021-09-11

**Authors:** Ryo Esumi, Tadashi Kaneko, Asami Ito, Yohei Ieki, Yoshiki Yamamoto, Ayako Nakajima, Hiroshi Imai

**Affiliations:** a Emergency and Critical Care Center, Mie University Hospital, Japan; b Department of Rheumatology, Center for Rheumatic Diseases, Mie University Hospital, Japan

**Keywords:** Heparin, Thrombus, Computed tomography

## Abstract

Antineutrophil cytoplasmic antibody-associated vasculitis (AAV) is sometimes complicated by diffuse alveolar hemorrhage (DAH), which may cause respiratory failure. Venovenous extracorporeal membrane oxygenation (VV-ECMO) without an anticoagulant because of hemorrhagic status, showed the effectiveness for severe respiratory failure by DAH with AAV. A 44-year-old woman developed DAH with bowel bleeding following the onset of AAV, with positive anti-proteinase-3 (PR3) antibodies. Although ventilator management could not support her respiratory status, VV-ECMO was performed. The patient was given immunosuppressive therapy comprising a steroid pulse, plasma exchange, and cyclophosphamide. After about 10 days of VV-ECMO and immunosuppressive therapy, VV-ECMO was withdrawn, and on day 12, ventilator support was stopped. Although a thrombus developed within the inferior vena cava (IVC), which required IVC filtration, the patient was discharged on day 51. VV-ECMO support was effective for treating DAH in this patient with new-onset AAV, which takes some time to achieve remission with immunosuppressive therapy.

## 1 Introduction

Antineutrophil cytoplasmic antibody-associated vasculitis (AAV) is a frequent complication of interstitial lung disease (ILD). It has been reported that 46%–71% of patients are positive for anti-myeloperoxidase (MPO) antibodies and 0–29% are positive for anti-proteinase-3 (PR3) antibodies. Besides AAV sometimes develop diffuse alveolar hemorrhage (DAH) as a complication [1]. In recent years, the 5-year survival rate of AAV has increased from 12% to 70%, but AAV as a complication of ILD is a risk factor for poor outcomes [2]. DAH occurs in 8%–36% of patients with AAV, and 57% of patients are positive for PR3 antibodies [3]. Moreover, 56% of patients with DAH and AAV require admission to an intensive care unit (ICU) for the treatment of respiratory failure using invasive or noninvasive ventilators [4]. Here, we report a patient with DAH complicated with AAV who was positive for PR3 antibodies. The patient developed severe respiratory failure and was unable to tolerate conventional ventilator support. Anticoagulant-free venovenous extracorporeal membrane oxygenation (VV-ECMO) was necessary to maintain her respiratory function.

## 2 Case presentation

**Fig. 1:**
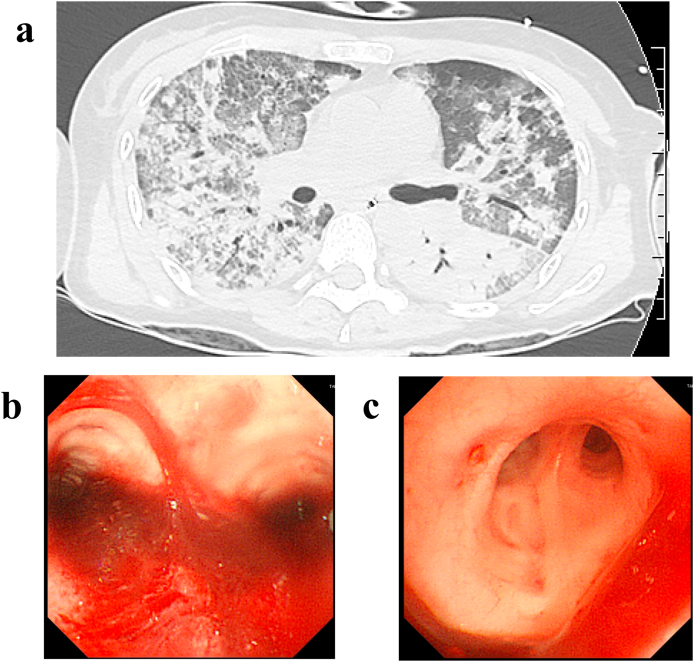
Imaging findings upon admission to our intensive care unit. (a) Computed tomography revealed diffuse consolidation and atelectasis due to diffuse alveolar hemorrhage affecting both lungs. (b) Bronchoscopy of the carina showing diffuse hemoptysis. (c) Bronchoscopy of the right upper bronchus showing an ulcer and hemoptysis.

A 44-year-old woman with hemoptysis, hematuria, and epistaxis, and positive for PR3 antibodies, was scheduled to be hospitalized for renal and nasal biopsies. However, because the patient's respiratory status worsened, tracheal intubation with ventilator support was necessary. Although two days of steroid pulse therapy (1 g/day methylprednisolone) was started, her respiratory status did not improve within 2 days, and the patient required ventilator support with nearly 100% oxygen. Therefore, the patient was transferred to the ICU at our hospital with a suspected diagnosis of new-onset DAH with AAV (Fig. 1). On admission to the ICU, her oxygen partial pressure was 67.8 mmHg and her carbon dioxide partial pressure was 46.3 mmHg while on 100% oxygen and positive end-expiratory pressure. Laboratory tests revealed the following: PR3 antibody titer, 28.2 IU/mL; white blood cell count, 16,100/μL; hemoglobin, 6.1 g/dL; platelet count, 187,000/μL; albumin, 2.1 g/dL; blood urea nitrogen, 23.7 mg/dL; creatinine, 0.44 mg/dL; aspartate aminotransferase, 34 U/L; alanine aminotransferase, 19 U/L; lactate dehydrogenase, 779 U/L; total bilirubin, 1.1 mg/dL; creatinine phosphokinase, 657 U/L; C-reactive protein, 9.67 mg/dL; prothrombin time, 70.5%; activated partial thromboplastin time, 23.6 s; as well as hematuria and proteinuria.

Anticoagulant-free VV-ECMO was initially started using a heparin-coated circuit and it was planned to introduce anticoagulant gradually. However, bowel bleeding occurred on day 2 of admission. Because a colonoscopy showed multiple ulcers, an anticoagulant was not used for almost the entire duration of VV-ECMO. An anticoagulant (nafamostat) was trialed on day 9, but it needed to be stopped due to recurrent bowel bleeding.

**Fig. 2:**
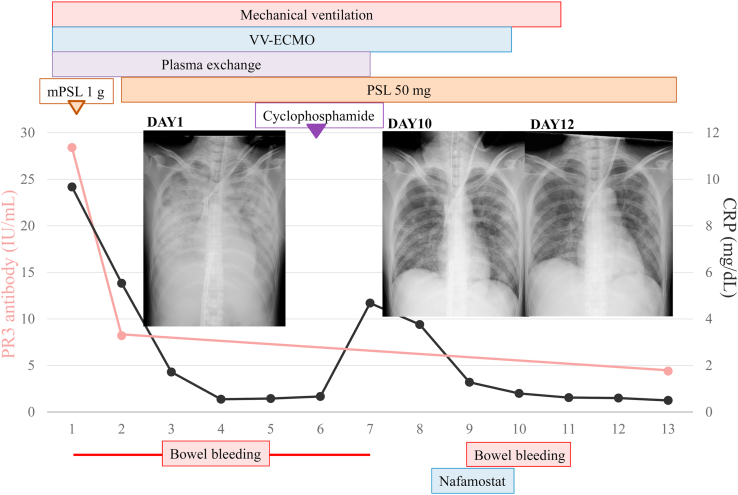
Clinical course. CRP = C-reactive protein; mPSL = methylprednisolone; PSL = prednisolone; PR3 = anti-proteinase-3; VV-ECMO = venovenous extracorporeal membrane oxygenation.

To treat AAV, the patient was given a steroid pulse for 1 day followed by 1.0 mg/kg/day of prednisolone, and daily plasma exchange (7 times in 8 days). Additionally, 500 mg of cyclophosphamide was infused on day 6 (Fig. 2). After starting VV-ECMO support, oxygen partial pressure was maintained more than 150 mmHg by VV-ECMO with 100% oxygen gas and 3.5–4.0 L/min flow. On day 11, bilateral diffuse infiltration shadow by DAH was decreased on chest X-ray, after checking more than 250 mmHg of P/F ratio with clamping ECMO oxygen gas (no oxygen), VV-ECMO was withdrawn. On day 12, P/F ratio showed more than 400 mmHg, therefore, ventilator support was stopped and the patient was extubated. Only one VV-ECMO circuit could be completed without anticoagulant within a 10-day course without circuit exchange. However, deep vein thrombosis in the inferior vena cava (IVC) was diagnosed by computed tomography on day 12, which required IVC filtration management for 17 days, afterwards there was no recurrence of thrombus.

The patient was transferred from the ICU to a general ward on day 13, and discharged from hospital on day 51, after four infusions of cyclophosphamide (500 mg) by every two weeks. After a total of six infusions of cyclophosphamide, AAV was relapsed and the patient was prescribed rituximab (500 mg) by every two weeks, started 71st day from discharge, combined with 0.5 mg/kg/day of prednisolone.

## 3 Discussion

In the present case, although steroid pulse therapy was started after the diagnosis of DAH with AAV, the patient's respiratory failure could not be managed with conventional ventilator support at a previous hospital. After being transferred to our hospital, plasma exchange, steroid pulse, and cyclophosphamide were started, and we also decided to use VV-ECMO support. It took about 10 days to treat her DAH and suppress hemorrhage, after which VV-ECMO support was successfully withdrawn.

Some papers have reported the use of VV-ECMO support for managing respiratory failure caused by DAH with AAV [5,6]. These papers showed that VV-ECMO could support the respiratory function until the initial immunosuppressive therapies had elicited a sufficient effect, especially in new-onset AAV cases. Prior cases were 13–65 years old, included males and females, and VV-ECMO support lasted 5–21 days. Our case required VV-ECMO support for 10 days, which is within the expected range for the immunosuppressive therapy to elicit clinical effects in new-onset AAV.

Gastrointestinal symptoms were reported in 7% of cases of AAV [7]. Our case experienced bowel bleeding and colonoscopy revealed multiple ulcers in her colon. This symptom is likely to be related to AAV and improved following immunosuppressive therapy. However, bowel bleeding and DAH precluded the use of an anticoagulant. Therefore, VV-ECMO was managed using a heparin-coated circuit, without an anticoagulant. A previous report described the use of VV-ECMO without or with a low dose of heparin for cases with a high risk of bleeding. The frequencies of thrombotic and bleeding complications did not differ from those of full-dose heparin [8]. In our case, to maintain the lifetime of anticoagulant-free VV-ECMO, we tried to maintain the pump flow above 4 L/min. Consequently, VV-ECMO could be continued for 10 days, but an IVC thrombus developed that required treatment.

In a single-center historical cohort, Cartin-Ceba et al. reported that DAH occurred in 8.3% of patients with AAV, who were then treated with plasma exchange, corticosteroid, rituximab, and/or cyclophosphamide. VV-ECMO support was not performed. The length of ICU stay was 6.1 days and 11% of patients died [4]. Because our patient received VV-ECMO support, her ICU stay was longer, at 13 days.

Reddy et al. reported the review article about ECMO support for DAH and diffuse alveolar damage. The review contained 38 cases with 84.2% of DAH, VV-ECMO for 73.7% and venoarterial-ECMO for 13.2%, median duration of ECMO support was 9.5 days, hospital mortality was 10.5% (VV-ECMO was 7.1%), ECMO circuit thrombus was occurred in 10.5% (rate of using anticoagulant was unknown) [9].

VV-ECMO support for DAH with AAV was successfully performed without an anticoagulant owing to the patient's risk of bleeding, although an IVC thrombus occurred. Although the length of ICU stay is expected to be longer, VV-ECMO support can be a useful strategy for severe DAH with AAV, especially for new-onset cases that require longer immunosuppressive therapy to achieve remission.

## 4 Conclusion

This case of DAH with AAV was treated with conventional immunosuppressive therapies combined with anticoagulant-free VV-ECMO support. This strategy could be useful for managing patients with severe respiratory failure, especially those with new-onset DAH with AAV, who have not received any specific therapies.

Author Declaration of No Conflict of Interest.

Authors have no financial or non-financial interest.

## Authors’ contributions

Both RE and TK have authorship contribution and wrote the first draft of the manuscript. All the authors revised and commented on the manuscript, and approved the final version.

## Author Declaration of No conflict of interest

Authors have no financial or non-financial interest.
